# Better antimicrobial resistance data analysis and reporting in less time

**DOI:** 10.1093/jacamr/dlac143

**Published:** 2023-01-18

**Authors:** Christian F Luz, Matthijs S Berends, Xuewei Zhou, Mariëtte Lokate, Alex W Friedrich, Bhanu Sinha, Corinna Glasner

**Affiliations:** Department of Medical Microbiology and Infection Prevention, University of Groningen, University Medical Center Groningen, Hanzeplein 1, 9713 GZ, Groningen, Netherlands; Department of Medical Microbiology and Infection Prevention, University of Groningen, University Medical Center Groningen, Hanzeplein 1, 9713 GZ, Groningen, Netherlands; Department of Medical Epidemiology, Certe Medical Diagnostics and Advice Foundation, Groningen, The Netherlands; Department of Medical Microbiology and Infection Prevention, University of Groningen, University Medical Center Groningen, Hanzeplein 1, 9713 GZ, Groningen, Netherlands; Department of Medical Microbiology and Infection Prevention, University of Groningen, University Medical Center Groningen, Hanzeplein 1, 9713 GZ, Groningen, Netherlands; Department of Medical Microbiology and Infection Prevention, University of Groningen, University Medical Center Groningen, Hanzeplein 1, 9713 GZ, Groningen, Netherlands; Department of Medical Microbiology and Infection Prevention, University of Groningen, University Medical Center Groningen, Hanzeplein 1, 9713 GZ, Groningen, Netherlands; Department of Medical Microbiology and Infection Prevention, University of Groningen, University Medical Center Groningen, Hanzeplein 1, 9713 GZ, Groningen, Netherlands

## Abstract

**Objectives:**

Insights about local antimicrobial resistance (AMR) levels and epidemiology are essential to guide decision-making processes in antimicrobial use. However, dedicated tools for reliable and reproducible AMR data analysis and reporting are often lacking. We aimed to compare traditional data analysis and reporting versus a new approach for reliable and reproducible AMR data analysis in a clinical setting.

**Methods:**

Ten professionals who routinely work with AMR data were provided with blood culture test results including antimicrobial susceptibility results. Participants were asked to perform a detailed AMR data analysis in a two-round process: first using their software of choice and next using our newly developed software tool. Accuracy of the results and time spent were compared between both rounds. Finally, participants rated the usability using the System Usability Scale (SUS).

**Results:**

The mean time spent on creating the AMR report reduced from 93.7 to 22.4 min (*P* < 0.001). Average task completion per round changed from 56% to 96% (*P* < 0.05). The proportion of correct answers in the available results increased from 37.9% in the first to 97.9% in the second round (*P* < 0.001). Usability of the new tools was rated with a median of 83.8 (out of 100) on the SUS.

**Conclusions:**

This study demonstrated the significant improvement in efficiency and accuracy in standard AMR data analysis and reporting workflows through open-source software. Integrating these tools in clinical settings can democratize the access to fast and reliable insights about local microbial epidemiology and associated AMR levels. Thereby, our approach can support evidence-based decision-making processes in the use of antimicrobials.

## Introduction

Antimicrobial resistance (AMR) is a global challenge in healthcare, livestock and agriculture, and the environment alike. The silent tsunami of AMR is already impacting our lives and the wave is constantly growing.^1,2^ One crucial action point in the fight against AMR is the appropriate use of antimicrobials. The choice and use of antimicrobials have to be integrated into a well-informed decision-making process and supported by antimicrobial and diagnostic stewardship programmes.^3,4^ Next to essential (setting-specific) guidelines on appropriate antimicrobial use, the information on AMR rates and antimicrobial use through reliable data analysis and reporting is vital. While data on national and international levels are typically easily accessible through official reports, local data insights are often lacking, difficult to establish, and their generation requires highly trained professionals. This is often further complicated by very heterogeneous data structures and information systems within and between different settings.^5,6^ Yet, decision-makers in the clinical context need to be able to access these important data in an easy and rapid manner. Incorrect data or data analyses could even lead to biased/erroneous empirical antimicrobial treatment policies.

To overcome these hurdles, we previously developed new approaches to AMR data analysis and reporting to empower any expert on any level working with or relying on AMR data.^7,8^ We aimed for reliable, reproducible and transparent AMR data analysis. In addition, we demonstrated the application of this software package to create interactive analysis tools for rapid and user-friendly AMR data analysis and reporting.^8^ Thereby, we could fill an important gap, defined by the lack of available (free and open-source) software tools that fulfil all requirements such as incorporation of (inter-)national guidelines or reliable reference data.

However, while the use of our approach in research has been demonstrated,^9–12^ the impact on workflows for AMR data analysis and reporting in clinical settings is pending. AMR data analysis and reporting are typically performed in clinical microbiology departments in hospitals, in microbiological laboratories, or as part of multidisciplinary antimicrobial stewardship activities.

In this study, we aimed to demonstrate and study the usability of our developed approach and its impact on clinicians’ workflows in an institutional healthcare setting. The approach aimed for better AMR data analysis and reporting in less time.

## Methods

The study was initiated at the University Medical Center Groningen (UMCG), a 1339 bed tertiary care hospital in the Northern Netherlands and performed across the UMCG and Certe (a regional laboratory) in the Northern Netherlands. It was designed as a comparison study to evaluate the efficiency, effectiveness and usability of a new AMR data analysis and reporting approach^7,8^ against traditional reporting.

### Study setup

**Figure 1.**
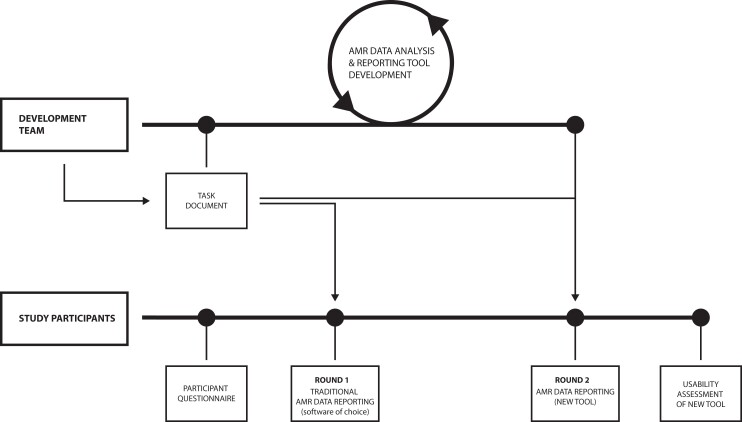
Study setup; the same AMR data were used along all steps and rounds.

The setup of the study is visualized in Figure 1 and explained in the following sections.

The study was based on a task document listing general AMR data analysis and reporting tasks (Table 1). This list served as the basis to compare effectiveness (solvability of each task for every user) and efficiency (time spent solving each task) of both approaches. Tasks were grouped into five related questions (further referred to as five tasks). A maximum amount of time per task (group) was defined for each task. The list of tasks including correct results is available in Appendix A1, available as Supplementary data at*JAC*Online.

**Table 1.** AMR data analysis and reporting tasks

Task	Task description	Maximum given time per task (min)^a^
1	Total number of blood culture sets per year	15
2a	Total number of positive blood culture sets per year	20
2b	Total number of negative blood culture sets per year	
3	Top 10 isolated microorganisms per year including isolate count (first isolates^b^)	20
4a	Resistance profile (S/I & R) in *Escherichia coli* (first isolates^b^) for selected antimicrobials	30
4b	Resistance profile (S/I & R) in *Klebsiella pneumoniae* (first isolates^b^) for selected antimicrobials	
4c	Resistance profile (S/I & R) in *Staphylococcus aureus* (first isolates^b^) for selected antimicrobials	
5a	Empirical treatment coverage for *E. coli* and *K. pneumoniae* (first isolates^b^ only for both) with a combination of cefuroxime and tobramycin	30
5b	Empirical treatment coverage for *E. coli* and *K. pneumoniae* (first isolates^b^ only for both) with a combination of amoxicillin + clavulanic acid + tobramycin OR amoxicillin + clavulanic acid + gentamicin	
5c	Empirical treatment coverage for *E. coli* and *K. pneumoniae* (first isolates^b^ only for both) with a combination of ceftriaxone + tobramycin OR ceftriaxone + gentamicin	

S = susceptible; I = susceptible, increased exposure; R = resistant.

a The maximum given time was the same for round 1 and round 2. If more time was spent than is stated here, this number was used as a maximum.

b CLSI, M39-A5.^13^

### AMR data

Data were collected retrospectively and permission was granted by the local ethical committee (METc 2014/530). Anonymized microbiological data were obtained from the Department of Medical Microbiology and Infection Prevention at the UMCG. The data consisted of 23 416 records from 18 508 unique blood culture tests that were taken between 1 January 2019 and 31 December 2019, which were retrieved from the local laboratory information system (LIS). The exemplified data structure is presented in Table 2.

**Table 2.** Raw data example

Patient ID	Date	Sample ID	Specimen	Microorganism	PEN	AMX	CXM
0001	8 March 2019	100	blood	esccol	R	I	S
0001	9 March 2019	101	blood	esccol	R	I	S
0002	8 March 2019	102	blood	staaur	R	S	—
0003	8 March 2019	103	blood	pseaer	R	R	R

S, susceptible; I, susceptible, increased exposure; R, resistant; PEN, penicillin; AMX, amoxicillin; CXM, cefuroxime; esccol, *E. coli*; staaur, *S. aureus*; pseaer, *Pseudomonas aeruginosa*.

### AMR data analysis, agile workflow and reporting

**Figure 2.**
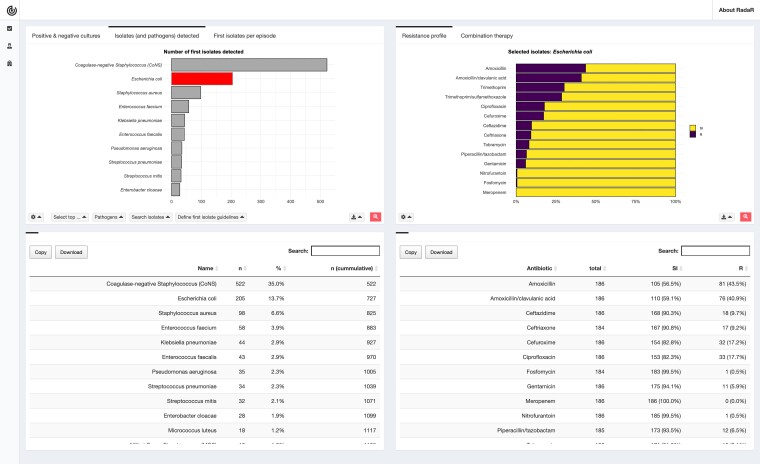
Interactive dashboard for AMR data analysis used in this study. This screenshot shows an overview of the tailored application built for this purpose. This web application is interactive and contains tabs and buttons to select, filter and present the underlying data.

We used our previously developed approach^7,8^ to create a customized browser-based AMR data analysis and reporting application. This application was used in this study and applied to the AMR data analysis and reporting tasks (Table 1). The development of the application followed an agile approach using scrum methodologies and involved two developers, a clinical microbiologist and an infection preventionist.^14^ Scrum is a framework for project management in which work is split into short-term achievable goals, which has been proven to help with the integration of roles and knowledge to a project. Also, in medical research, scrum has been shown to efficiently empower quality, technology and implementation in scientific projects.^15^ Very recently, researchers showed that scrum can complement established quality assurance and software engineering practices by promoting ‘a social environment that is conducive to creating high-quality software’.^16^ The resulting application was designed as an interactive web-browser based dashboard (Figure 2). The prepared dataset was already loaded into the system and interaction with the application was possible through any web browser.

Analyses of AMR data in the participating departments is a regular task and comprises data from within departments (from and within local electronic health record systems) and across settings (e.g. in multicentre studies). Data analysis tools (in this manuscript: traditional tools) for regular analysis available to study participants were tools such as IBM SPSS Statistics software (SPSS) and Microsoft Excel to allow for raw data processing (in particular in multicentre approaches to overcome interoperability issues).

### Study participants

Participants in this study were recruited from the departments of Medical Microbiology, Critical Care Medicine and Paediatrics, to reflect heterogeneous backgrounds of healthcare professionals working with AMR data. Members of the development team did not take part in the study.

### Study execution and data

First, study participants were asked to fill in an online questionnaire capturing their personal backgrounds, demographics, software experience and experience in AMR data analysis and reporting. Next, participants were provided the task document together with the AMR data (csv or xlsx format). The participants were asked to perform a comprehensive AMR data report following the task document using their software of choice (round 1). Task results and information on time spent per task were self-monitored in a structured report form. Lastly, participants repeated the tasks using the new AMR data analysis and reporting application (round 2). Task results and information on time spent per task were again self-monitored in a structured report form. Round 2 was evaluated using a second online questionnaire. The study execution process is illustrated in Figure 1.

### Evaluation and study data analysis

**Figure 3.**
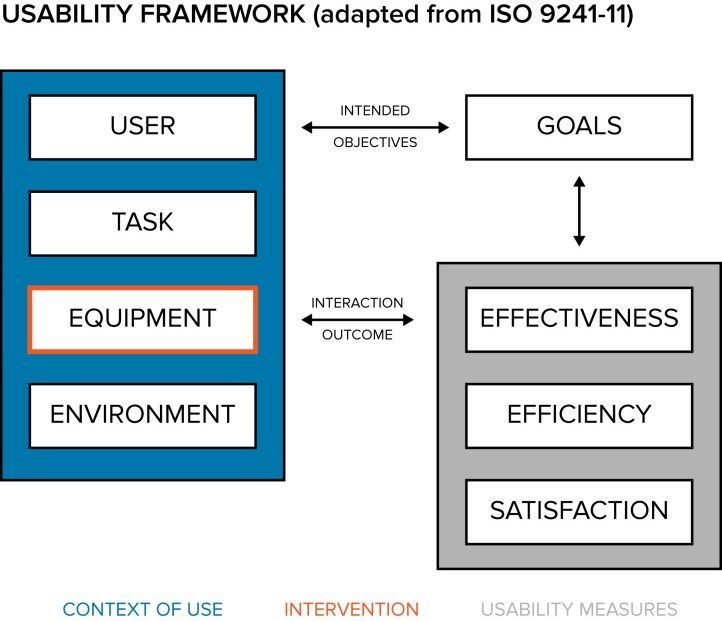
Usability framework based on ISO 9241-11.

The utility of the new AMR data analysis and reporting application was evaluated according to ISO 9241-11:2018.^17^ This international standard comprises several specific metrics to quantify the usability of a tool with regard to reaching its defined goals (Figure 3). In this study the goal was a comprehensive AMR data report and comprised several tasks. The equipment was the focus of this study (traditional AMR data analysis and reporting approach versus newly developed AMR data analysis and reporting approach).

The three ISO standard usability measures were defined as follows in this study. Effectiveness was determined by degree of task completion coded using three categories: (1) completed; (2) not completed (task not possible to complete); and (3) not completed (due to given time limits). In addition, effectiveness was assessed by the variance in the task results stratified by study round. Deviation from the correct results was measured in absolute percent from the correct result. Efficiency was determined by timing each individual task. Time on task started when the user started performing the task, all data was loaded and the chosen analysis software was up and running. Time on task ended when the task reached one of the endpoints, as described above. The mean time for each task and the mean total time for the complete report across users was calculated. Statistically significant difference was tested using paired Student’s *t*-test. Outcomes of tests were considered statistically significant for *P* < 0.05. All analyses were performed in R.^18^ Accuracy of the reported results per task and round were studied by calculating the deviation of the reported result in absolute percent from the correct result. Satisfaction was measured using the System Usability Scale (SUS), a 10-item Likert scale with levels from 1 (strongly disagree) to 5 (strongly agree, see Appendix A3).^19^

## Results

### Study participants

In total, 10 participants were recruited for this study. Most participants were clinical microbiologists (in training) (70%). The median age of the participant group was 40.5 years with a median field experience of 8.0 years. The relevance of AMR data as part of the participants’ job was rated with a median of 5.0 (scale 1–5). AMR data analysis was part of 60% of all participants’ jobs. Participants reported to be experienced in interpreting AMR data structures (median 5.0, scale 1–5). Participants were less experienced in epidemiological data analysis (median 3.0, scale 1–5). All participant characteristics are summarized in Table 3.

**Table 3.** Study participant characteristics

Characteristics	Overall (*n* = 10)
Age (years), median (min–max)	40.5 (32.0–61.0)
Working experience (years), median (min–max)	8.00 (1.00–22.0)
Job description, *n* (%)	
Infection preventionist	1 (10.0)
Intensivist	1 (10.0)
Clinical microbiologist	4 (40.0)
Paediatrician	1 (10.0)
Resident clinical microbiology	3 (30.0)
Worked with AMR data before, *n* (%)	
No	1 (10.0)
Yes	9 (90.0)
Relevance of AMR data as part of the job (1 = not relevant at all; 5 = very relevant), median (min–max)	5.00 (3.00–5.00)
AMR data analysis as part the job, *n* (%)	
No	4 (40.0)
Yes	6 (60.0)
Familiarity with AMR data structure (1 = not familiar at all; 5 = expert)	
Median (min–max)	4.00 (1.00–5.00)
Missing, *n* (%)	1 (10.0)
Experience in interpreting AMR data (e.g. antibiograms) (1 = no experience; 5 = very experienced), median (min–max)	5.00 (3.00–5.00)
Experience in epidemiological data analysis (1 = no experience; 5 = very experienced), median (min–max)	3.00 (2.00–5.00)
Experience in working with AMR data (1 = no experience; 5 = very experienced), median (min–max)	3.50 (1.00–5.00)

**Figure 4.**
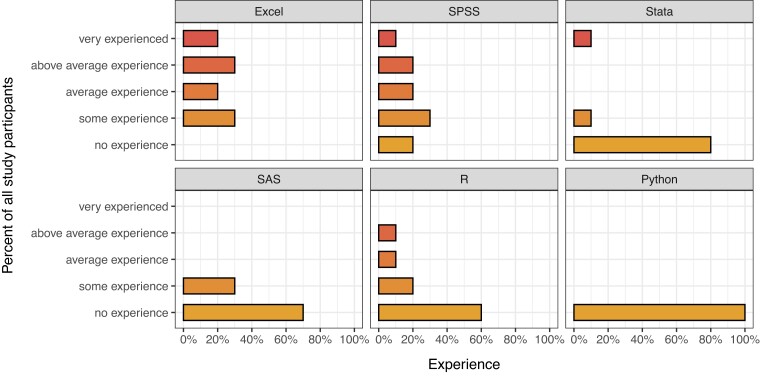
Data analysis software experience reported by study participants.

The participants reported a diverse background in software experience for data analysis, with most experience reported for Microsoft Excel (Figure 4).

### Effectiveness and accuracy

**Figure 5.**
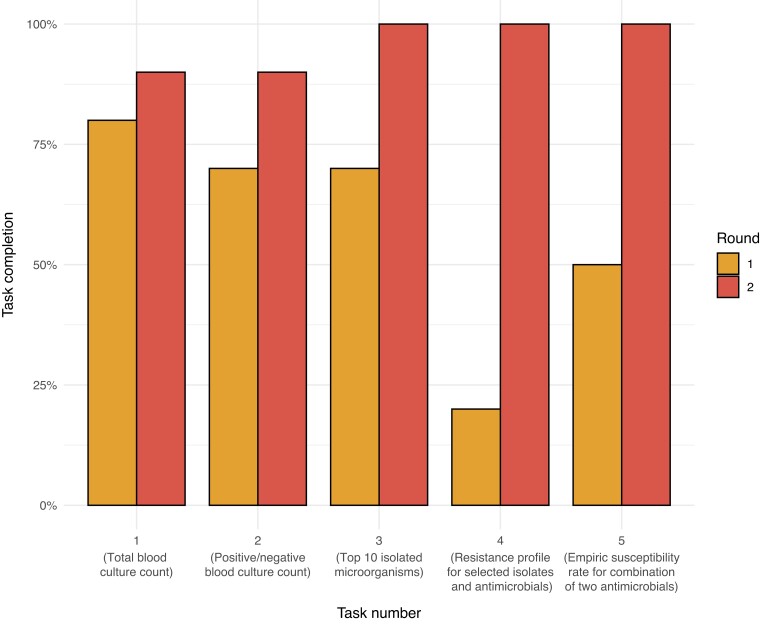
Task completion in percentage by task number and round. The task rounds are plotted along the *x*-axis with their brief description. For each task the difference in task completion was compared between round 1 and round 2.

Average task completion between the first round and the second round changed from 56% (SD: 23%) to 96% (SD: 6%) (*P* < 0.05). Task completion per question and round is displayed in Figure 5. Variation in responses for each given task showed significant differences between the first and second round.

**Figure 6.**
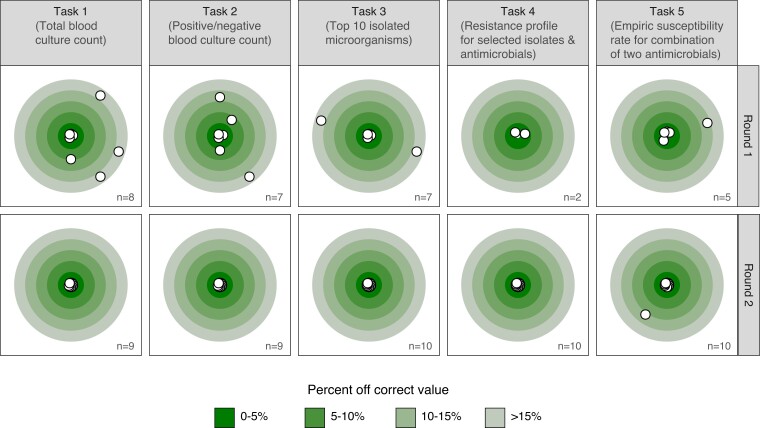
Deviation from the correct result by task and round in absolute percent from correct result. Only completed tasks (*n*) are shown.

Figure 6 shows the deviation in absolute percent from the correct results from the correct result per round and task. The proportion of correct answers in the available results increased from 38% in the first round to 98% in the second round (*P* < 0.001). A subanalysis of species-specific results for task 3 round 1 is available in Appendix A3.

### Efficiency

**Figure 7.**
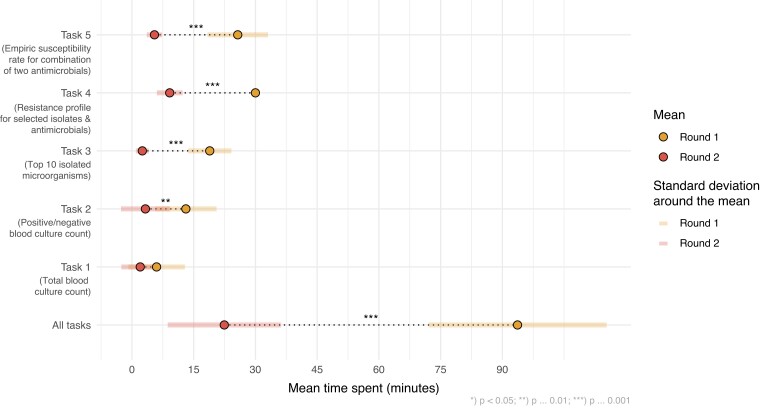
Mean time spent per task in minutes in each round. Statistical significance was tested using two-sided paired *t*-tests. All results were included irrespective of correctness of the results.

Overall, the mean time spent per round was significantly reduced from 93.7 (SD: 21.6) to 22.4 (SD: 13.7) min (*P* < 0.001). Significant time reduction was observed for tasks 2–5 (Figure 7). Analyses were further stratified to compare efficiency between participants who reported AMR data analysis as part of their job versus not part of their job. No significant time difference for completing all tasks was found between the groups. However, in both groups the overall time for all tasks significantly decreased between rounds, on average by 70.7 min (*P* < 0.001) in the group reporting AMR data analysis as part of their job and by 72.1 min (*P* = 0.01) in the group not reporting AMR data analysis as part of their job.

### Satisfaction

Participants rated the usability of the new AMR reporting tool using the SUS, which takes values from 0 to 100 (Appendix A2). This resulted in a median of 83.8 on the SUS.

## Discussion

This study demonstrates the effectiveness, efficiency and accuracy of using open-source software tools to improve AMR data analysis and reporting. We applied our previously developed approach to AMR data analysis and reporting^7,8^ in a clinical scenario and tested these tools with study participants working in the field of AMR. Comparing traditional reporting tools with our newly developed reporting tools in a two-step process, we demonstrated the usability and validity of our approach. Based on a five-item AMR data analysis and reporting task list and the provided AMR data, study participants reported significantly less time spent on creating an AMR data report (on average 93.7 versus 22.4 minutes; *P* < 0.01). Task completion increased significantly from 56% to 96%, which indicates that with traditional reporting approaches common questions around AMR are hard to answer in a limited time. The accuracy of the results greatly improved using the new approach, implicating that erroneous answers are more common when relying on non-AMR-specific traditional software solutions. The usability of our approach was rated with a median of 83.8 on the SUS. The SUS is widely used in usability assessments of software solutions. A systematic analysis of more than 1000 reported SUS scores for web-based applications across different fields has found a mean SUS score of 68.1.^20^ The results thus demonstrate good usability of our approach.

The task list used in this study reflects standard AMR reporting tasks. More sophisticated tasks, such as the detection of multidrug resistance according to (inter-)national guidelines were not included. However, these analyses are vital in any setting but restrained since the required guidelines are not included in traditional tools (e.g. Microsoft Excel, SPSS etc.). Notably, the underlying software used in this study^7^ does provide methods to easily incorporate (inter-)national guidelines such as the definitions for (multi)drug resistance and country-specific (multi)drug-resistant organisms. The increase in task completion rate and accuracy of the results demonstrated that our tools empower specialists in the AMR field to generate reliable and valid AMR data reports. This is important as it enables detailed insights into the state of AMR on any level. These insights are often lacking. Our approach could fill this gap by democratizing the ability for reliable and valid AMR data analysis and reporting.

This need is exemplified in the worrisome heterogeneity of the reporting results using traditional AMR reporting tools in round 1. Only 37.9% of the results in round 1 were correct. Together with a task completion rate of 56%, this demonstrates that traditional tools are not suitable for AMR reporting. The inability of working in reproducible and transparent workflows further aggravates reporting with traditional tools. All participants in the study should be able to produce standard AMR reports and 90% indicated that they worked with AMR data before. Sixty percent reported AMR data analysis to be part of their job, but no efficiency difference between groups was found. Our results show that AMR data analysis and reporting is challenging and can be highly error-prone. But an approach such as the one we developed can lead to correct results in a short time while being reproducible and transparent.

Our approach was inspired by others not in the AMR field that describe the use of reproducible open-source workflows in ecology.^21^ We found that open-source software enables the transferability of methodological approaches across research fields. This transfer is a great example of the strength in the scientific community when working interdisciplinarily and sharing reliable and reproducible workflow.

This study also has limitations. Only 10 study participants were recruited. Although low participant numbers are frequently observed in usability studies and reports show that only five participants suffice to study the usability of a new system, a larger sample size would be desirable.^22–26^ In addition, other evaluation methods (e.g. ‘think aloud’ method) beyond the single use of the SUS would further improve insights in the usability of our approach but were not possible in the study setting.^27^ Although the introduction of new AMR data and reporting tools made use of an already available approach, implementation still requires staff experienced in R. Reporting requirements also differ per setting and tailor-made solutions incorporating different requirements are needed.

The present study shows that answering common AMR-related questions is a burden for professionals working with data. However, answers to such questions are the requirement to enable hospital-wide monitoring of AMR levels and setting-specific treatment policies. It is thus of utmost importance that reliable results of AMR data analyses are ensured to avoid imprecise and erroneous results that could potentially be harmful to patients. We show that traditional reporting tools and applications are not equipped for conducting microbiology epidemiological analyses and seem unfit for this task—even for the most basic AMR data analyses. To fill this gap, we have developed new tools for AMR data analysis and reporting. In this study, we demonstrated that these tools can be used for better AMR data analysis and reporting in less time.

## Supplementary Material

dlac143_Supplementary_Data

## Supplementary data

Appendices A1–A3 are available as Supplementary data at*JAC*Online.
