# Supplementary material for: Better antimicrobial resistance data analysis and reporting in less time
Source: JAC Antimicrob Resist. 2023 Jan 18;5(1):dlac143. doi: 10.1093/jacamr/dlac143 (PMC9847555; doi:10.1093/jacamr/dlac143)
Supplement: dlac143_Supplementary_Data [file dlac143_supplementary_data.docx]

# Supplementary Material

## Appendix A1: Task lists including correct results

**Table A1.** AMR data analysis and reporting tasks with correct results

| Task | Task description | Correct result | |
| --- | --- | --- | --- |
| 1 | Total number of blood culture sets per year | 18,468 | |
| 2a | Total number of positive blood culture sets per year | 2,473 | |
| 2b | Total number of negative blood culture sets per year | 15,995 | |
| 3 | Top ten isolated microorganisms in blood cultures per year including isolate count (first isolates) |  | |
|  | CoNS | 522 | |
|  | *Escherichia coli* | 205 | |
|  | *Staphylococcus aureus* | 98 | |
|  | *Enterococcus faecium* | 58 | |
|  | *Klebsiella pneumoniae* | 44 | |
|  | *Enterococcus faecalis* | 43 | |
|  | *Pseudomonas aeruginosa* | 35 | |
|  | *Streptococcus pneumoniae* | 34 | |
|  | *Streptococcus mitis* | 32 | |
|  | *Enterobacter cloacae* | 28 | |
| 4 | Resistance profile (S/I & R) in selected isolates (first isolates) found in blood cultures for selected antimicrobials |  |  |
| A | *Escherichia coli* | S/I (%) | R (%) |
|  | Amoxicillin | 56.4 | 43.5 |
|  | Amoxicillin /clavulanic acid | 59.1 | 40.9 |
|  | Piperacillin /tazobactam | 93.5 | 6.5 |
|  | Cefuroxime | 82.8 | 17.2 |
|  | Ceftriaxone | 90.8 | 9.2 |
|  | Ceftazidime | 90.3 | 9.7 |
|  | Meropenem | 100.0 | 0.0 |
|  | Ciprofloxacin | 82.3 | 17.7 |
|  | Gentamicin | 94.1 | 5.9 |
|  | Tobramycin | 91.9 | 8.1 |
|  | Fosfomycin | 99.5 | 0.5 |
|  | Trimethoprim | 69.9 | 30.1 |
|  | Trimethoprim/sulfamethoxazole | 71.5 | 28.5 |
|  | Nitrofurantoin | 99.5 | 0.5 |
| B | *Klebsiella pneumoniae* | S/I (%) | R (%) |
|  | Amoxicillin | 0.0 | 100.0 |
|  | Amoxicillin /clavulanic acid | 75.6 | 24.4 |
|  | Piperacillin /tazobactam | 87.8 | 12.2 |
|  | Cefuroxime | 82.9 | 17.1 |
|  | Ceftriaxone | 85.4 | 14.6 |
|  | Ceftazidime | 85.4 | 14.6 |
|  | Meropenem | 100.0 | 0.0 |
|  | Ciprofloxacin | 87.8 | 12.2 |
|  | Gentamicin | 97.6 | 2.4 |
|  | Tobramycin | 92.7 | 7.3 |
|  | Fosfomycin | 80.5 | 19.5 |
|  | Trimethoprim | 85.4 | 14.6 |
|  | Trimethoprim / sulfamethoxazole | 90.2 | 9.8 |
|  | Nitrofurantoin | 0.0 | 100.0 |
| C | *Staphylococcus aureus* | S/I (%) | R (%) |
|  | Penicillin | 24.7 | 75.3 |
|  | Flucloxacillin | 96.6 | 3.4 |
|  | Gentamicin | 97.7 | 2.3 |
|  | Erythromycin | 85.4 | 14.6 |
|  | Clindamycin | 89.9 | 10.1 |
|  | Doxycycline | 97.8 | 2.2 |
|  | Linezolid | 100.0 | 0.0 |
|  | Trimethoprim / sulfamethoxazole | 94.4 | 5.6 |
|  | Rifampicin | 98.9 | 1.1 |
| 5 | Empiric susceptibility rate for selected isolates (first isolates) found in blood cultures with a combination of selected antimicrobials | S/I (%) | |
| A | *Escherichia coli* |  | |
|  | Cefuroxime & tobramycin | 96.8 | |
|  | *Klebsiella pneumoniae* |  | |
|  | Cefuroxime & tobramycin | 92.7 | |
| B | *Escherichia coli* |  | |
|  | Amoxicillin / clavulanic acid & tobramycin | 93.0 | |
|  | Amoxicillin / clavulanic acid & gentamicin | 95.2 | |
|  | *Klebsiella pneumoniae* |  | |
|  | Amoxicillin / clavulanic acid & tobramycin | 92.7 | |
|  | Amoxicillin / clavulanic acid & gentamicin | 97.6 | |
| C | *Escherichia coli* |  | |
|  | Ceftriaxone & tobramycin | 97.3 | |
|  | Ceftriaxone & gentamicin | 98.4 | |
|  | *Klebsiella pneumoniae* |  | |
|  | Ceftriaxone & tobramycin | 92.7 | |
|  | Ceftriaxone & gentamicin | 97.6 | |

## Appendix A2: System Usability Scale (SUS)

1. I think that I would like to use this system frequently.
2. I found the system unnecessarily complex.
3. I thought the system was easy to use
4. I think that I would need the support of a technical person to be able to use this system.
5. I found the various functions in this system were well integrated.
6. I thought there was too much inconsistency in this system.
7. I would imagine that most people would learn to use this system very quickly.
8. I found the system very cumbersome to use.
9. I felt very confident using the system.
10. I needed to learn a lot of things before I could get going with this system.

(Each item with levels: 1 = strongly disagrees to 5 = strongly agrees)

Scores for individual items are not meaningful on their own. To calculate the SUS score, the score contributions from each item must be summed. Each item’s score contribution ranges from 0 to 4. For items 1, 3, 5, 7, and 9 the score contribution is the scale position minus 1. For items 2, 4, 6, 8, and 10, the contribution is 5 minus the scale position. The sum of the scores is multiplied by 2.5 to obtain the SUS.

## Appendix A3: Usability framework based on ISO 9241-11.

##

## Appendix A4: Data analysis software experience reported by study participants.

## Appendix A5: Task 3 sub-analysis

Task 3 asked participants to identify the ten most frequent species in the provided data set, while correcting for multiple occurrences of a species within a patient. Figure A3 illustrates the deviation from the correct result in the first round (traditional AMR reporting) per species. For this analysis also incomplete results were included (i.e., task not completed but some results provided).

**
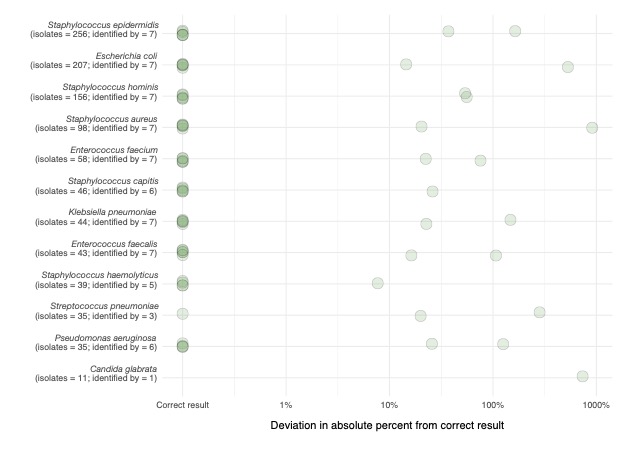
**

**Figure A3.** Results from task 3 in round 1. Deviation in absolute percent from the correct result per identified species. Also, incomplete data from participants was used in this analysis (i.e., task not completed but some results given). The correct number per species is given in addition to the number provided answers.
